# Alternative *trans*-splicing of *Caenorhabditis elegans sma-9/schnurri *generates a short transcript that provides tissue-specific function in BMP signaling

**DOI:** 10.1186/1471-2199-11-46

**Published:** 2010-06-17

**Authors:** Jianghua Yin, Ling Yu, Cathy Savage-Dunn

**Affiliations:** 1 Department of Biology, Queens College, and Biochemistry PhD Program, the Graduate School and University Center, the City University of New York, Flushing, NY 11367, USA

## Abstract

**Background:**

Transcription cofactors related to *Drosophila *Schnurri facilitate the transcriptional programs regulated by BMP signaling in *C. elegans*, *Drosophila*, Xenopus, and mouse. In different systems, Schnurri homologs have been shown to act as either agonists or antagonists of Smad function, and as either positive or negative regulators of transcription. How Schnurri proteins achieve this diversity of activities is not clear. The *C. elegans sma-9/schnurri *locus undergoes alternative splicing, including an unusual *trans*-splicing event that could generate two non-overlapping shorter transcripts.

**Results:**

We demonstrate here that the shorter transcripts are expressed *in vivo*. Furthermore, we find that one of the short transcripts plays a tissue-specific role in *sma-9 *function, contributing to the patterning of male-specific sensory rays, but not to the regulation of body size. Based on previous results, we suggest that this transcript encodes a C-terminal SMA-9 isoform that may provide transcriptional activation activity, while full length isoforms may mediate transcriptional repression and/or activation in a context-dependent manner.

**Conclusion:**

The alternative *trans*-splicing of *sma-9 *may contribute to the diversity of functions necessary to mediate tissue-specific outputs of BMP signaling.

## Background

The transforming growth factor β (TGFβ) superfamily comprises a large number of secreted peptide growth factors that have major regulatory effects on cell growth and differentiation [1,2]. Members of this superfamily include the TGFβs, the prototypes of the superfamily; the bone morphogenetic proteins (BMPs); and other members such as activin, inhibin, and Nodal. TGFβ superfamily ligands bind to a heteromeric receptor complex at the cell surface. This complex contains two related transmembrane serine/threonine kinases, the type I and type II receptors [3,4]. Signaling downstream of the receptors is mediated by the Smad proteins, which shuttle between the cytoplasm and nucleus to regulate target gene expression [5]. Type I receptors directly activate receptor-regulated Smads (R-Smads) by phosphorylation at C-terminal SXS sequences [6,7]. Phosphorylation of R-Smads promotes heterotrimeric complex formation with Co-Smads and accumulation in the nucleus to regulate gene transcription [7-11]. In mammals, five R-Smads are present: two (Smad2,3) that transduce TGFβ/activin/Nodal signals and three (Smad1,5,8) that transduce BMP signals. Strikingly, the TGFβ family ligands far outnumber the Smads available for signal transduction. Furthermore, many of these ligands are capable of eliciting diverse context-dependent responses. Thus, Smad complexes must be capable of mediating multiple diverse outcomes. It is thought that Smad complexes rely in part on transcription cofactors for appropriate regulation of target genes.

In the nematode *Caenorhabditis elegans*, the BMP-related factor DBL-1 regulates body size and male tail morphogenesis via a conserved receptor/Smad signaling pathway [12]. Using a genetic approach to uncover components of this pathway, we previously identified *sma-9*, a gene that is required for the body size and male tail patterning functions of DBL-1 [13]. *sma-9 *is also required for patterning of the mesodermal lineage, in which it acts antagonistically to the DBL-1 pathway [14]. *sma-9 *is predicted to encode multiple protein products homologous to *Drosophila *Schnurri, a large zinc finger transcription cofactor that functions in Dpp/BMP signaling [15,16]. Analysis of *sma-9 *therefore provides the opportunity to elucidate the requirements for transcription cofactor function in an *in vivo *model system during the course of development. In addition to *Drosophila *Schnurri and *C. elegans *SMA-9/Schnurri, three vertebrate Schnurri homologs have been identified. These bind the κB-binding site and function in T cell development [17-19]. Notably, vertebrate Schnurri homologs have more recently been demonstrated to mediate transcriptional regulation downstream of BMP and TGFβ ligands, indicating a conserved role for these family members in TGFβ signal transduction [20-22].

The *sma-9 *open reading frame (ORF) predicted from genomic sequence encodes a protein of 2170 aa consisting of an N-terminal Gln-rich domain (encoded by predicted exons 1-3) and a C-terminal domain containing seven Zn fingers (in predicted exons 12-22). The sequencing of cDNA clones, however, revealed at least eight different mRNA species with alternative protein coding regions [13]. Interestingly, like *sma-9*, human Shn-1 and Shn-3 genes undergo alternative splicing [23,24], but the functional consequences of this processing have not been addressed. The *sma-9 *cDNA clones were classified based on their potential to code for the seven C-terminal Zn fingers: class I encodes all seven Zn fingers (ZF1-7), class II encodes the first pair and the triplet of Zn fingers (ZF1-5), and class III encodes only the first pair (ZF1-2). To determine whether these domains have different functions *in vivo*, Foehr et al. created genomic/cDNA hybrid constructs and tested their ability to rescue the body size and mesodermal patterning defects of *sma-9 *mutants [14]. They found that constructs encoding class I and class II C-termini were capable of rescuing both phenotypes, while the class III construct was not, suggesting that the presence of the Zn finger triplet is critical for function in body size and mesodermal lineage regulation.

**Figure 1:**
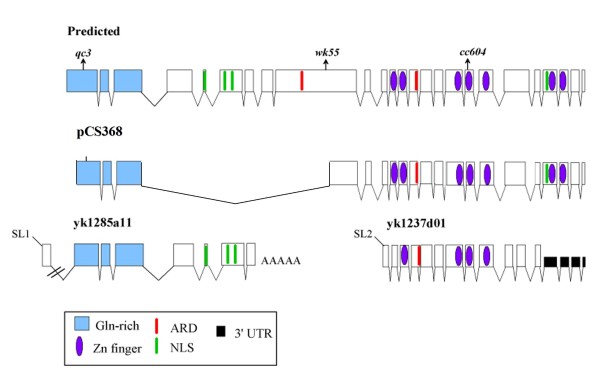
**Structure of *sma-9 *transcript variants**. The predicted *sma-9 *intron-exon structure is shown above. Arrows mark positions of nonsense codons in three *sma-9 *alleles. pCS386 is the most complete cDNA variant isolated to date. The two short variants represented by cDNA clones *yk1285a11 *(A11) and *yk1237d01 *(D01) are shown below. ARD: acidic residue-rich domain; NLS: nuclear localization signal.

Since *sma-9 *is a large gene, most of the existing cDNA clones contain incomplete coding sequences that are missing the 5' end of the gene. Two cDNA clones, *yk1285a11 *and *yk1237d01*, however, are notably different (Figure 1). Although these two cDNA clones are less than half the length of the predicted transcript, both of them have all of the hallmarks of a full-length cDNA: *trans*-spliced leader sequence, poly(A) tail, and a complete ORF. In *C. elegans*, many transcripts are processed at the 5' end by *trans-*splicing, which results in the addition of a 22-nucleotide splice leader sequence, SL1 or SL2 [25]. The mechanism of *trans*-splicing is similar to that of *cis-*splicing (intron removal), except that the splice donor sequences are provided by the SL1 and SL2 genes, rather than being contained within the context of the individual gene [25]. About half of all *C. elegans *genes are subjected to SL1 *trans*-splicing at the 5' end. A smaller subset of *C. elegans *genes are transcribed in polycistronic operons. In an operon, the 5'-most gene generally receives the SL1 splice leader. The individual downstream genes in an operon are then separated via SL2 *trans*-splicing to the 5' ends of each of the downstream genes. SL2 *trans*-splicing is accompanied by polyadenylation to create the 3' end of the neighboring gene upstream. *yk1285a11 *contains the SL1 splice leader and sequences from predicted exons 1-7. *yk1237d01 *contains the SL2 splice leader and sequences from predicted exons 11-25. As previously noted [13], the putative intercistronic region contains features reminiscent of the identified signals for *trans*-splicing, including a U-rich sequence, but is atypically long [26,27]. Thus, the structures of the *sma-9 *cDNA clones *yk1285a11 *and *yk1237d01 *suggest that they may represent two transcripts processed from a single longer transcript by SL2 *trans*-splicing and polyadenylation as normally occurs in a *C. elegans *polycistronic operon.

Since each of these *trans-*spliced short *sma-9 *transcripts was only represented by a single cDNA clone, we could not be certain that the clones were not due to cloning artifacts or a rare spurious event. Furthermore, our previous analysis did not address whether these short transcripts provide any function required for DBL-1 signal transduction. We therefore address here several remaining questions about these predicted transcripts. First, can we verify that the mRNA variants represented by *yk1285a11 *and *yk1237d01 *are expressed *in vivo*, rather than being artifacts produced during cDNA library construction? If so, then how is the expression of these variants regulated? Finally, do these variants show functional differences between each other and/or relative to full-length transcripts *in vivo*? In this work, we will refer to the splice variants represented by clones *yk1285a11 *(5') and *yk1237d01 *(3') as A11 and D01, respectively.

## Results

### Isolation of cDNAs spanning upstream and downstream regions

The *sma-9 *ORF predicted from genomic sequence is encoded by 25 exons ([13]; Figure 1). Previously characterized cDNA clones, however, contained only a subset of these 25 exons and, in particular, none of the existing cDNA clones spanned a region including both the N-terminal Gln-rich domain and the C-terminal Zn finger domains. We therefore used primers in exons 1 and 25 with the potential to generate nearly full-length cDNA inserts by RT-PCR. After RT-PCR, the clones with the longest inserts were selected for sequencing. The most complete cDNA clone isolated by this approach was pCS368, in which exons 4-8 and part of exon 9 are spliced out (Figure 1). This transcript form is reminiscent of a form identified by the ORFeome project, in which exon 3 becomes spliced to exon 20 [28]. These results thus support the existence of *sma-9 *transcripts capable of encoding both the Gln-rich and the Zn finger domains. Additional support derives from RT-PCR data using exon 9 primers (see below).

### Expression of sma-9 variant transcripts

**Figure 2:**
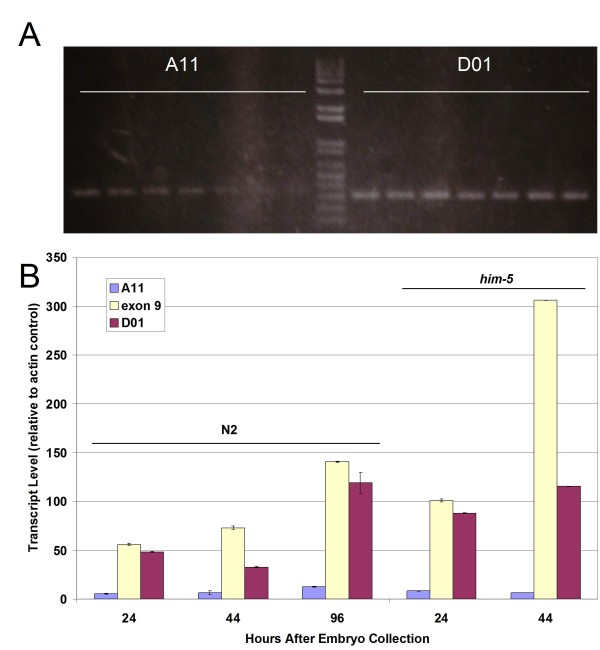
**Expression of *sma-9 *transcript variants**. **A**. Detection of A11 and D01 transcripts using variant-specific RT-PCR over a gradient of annealing temperatures (left to right: 50°C - 55°C). The expected size of the PCR product is 330 bp for A11 and 300 bp for D01. **B**. Developmental profile of *sma-9 *transcript expression levels, as determined by qRT-PCR. Data are shown as transcript abundance relative to *act-1 *actin gene control, and error bars show standard deviation. See Table 1 for quantitation of transcript abundance relative to exon-9-containing control.

We next sought to obtain evidence that the short transcripts represented by cDNA clones *yk1285a11 *and *yk1237d01 *(A11 and D01) are expressed *in vivo*, rather than cDNA artifacts. To address this question, we needed an approach to distinguish these short transcripts from full-length transcripts containing the same internal sequences. Unfortunately, we were unable to detect *sma-9 *mRNAs by Northern blot (data not shown). Instead, we used the unique termini of the short transcripts to design variant-specific primer pairs for RT-PCR. For A11 we employed an oligo-dT-containing reverse primer anchored by two nucleotides from *sma-9 *exon 7 with a gene-specific internal forward primer; for D01 we used an SL2-containing primer anchored by two nucleotides from *sma-9 *exon 11 with a gene-specific internal reverse primer. To determine empirically whether these primer pairs are specific for A11 and D01, we performed RT-PCR using a gradient of annealing temperatures (50°C - 55°C) and analyzed the products (Figure 2A). In both cases, the primer pairs amplified a single band of the correct size (330 bp for A11 and 300 bp for D01), indicating that the primers specifically detect the desired transcripts. Since the higher annealing temperatures resulted in reduced yield, we used 50°C for all subsequent analyses. Based on these results, we conclude that transcripts polyadenylated downstream of exon 7 and transcripts containing the SL2 splice leader sequence upstream of exon 11 are represented in the pool of *sma-9 *mRNAs *in vivo*.

The initial RT-PCR experiments were performed non-quantitatively on mixed-stage RNA preparations. We subsequently used real-time quantitative RT-PCR (qRT-PCR) to determine the relative expression levels of *sma-9 *variant transcripts and whether they are developmentally or sex-specifically regulated. Since we expect full-length transcripts, but not the short transcripts, to contain sequences from exon 9, we used exon 9 internal primers to determine the expression levels of potentially full-length transcripts and calculated the abundance of the short transcripts relative to exon-9-containing transcripts. Three developmental time points were assayed: 24 hr (first larval stage - L1), 44 hr (approximately L3), and 96 hr (adults). We find that exon-9-containing transcript levels increase during larval development and are highest in adulthood (Figure 2B). D01 transcripts accumulate in wild-type strain N2 at 45% - 85% of the level of exon-9-containing transcripts at all developmental time points examined, while A11 transcripts accumulate at approximately 10% of the level of exon-9-containing transcripts (Figure 2B; Table 1). If A11 and D01 are produced concurrently by *trans-*splicing of full-length transcripts, then the reduced accumulation of A11 may be due to differential stability of the transcripts. One possible mechanism for reduced stability of the A11 transcript is the unusual location of the termination codon TAA, in which the terminal adenines are introduced during polyadenylation and are contiguous with the poly(A) tail. To test for sex-specific expression of these transcripts, *him-5 *(high incidence of males) mutant populations that contain both hermaphrodites and males were compared to wild-type N2 hermaphrodite populations. In *him-5 *populations, the full-length and the D01 transcripts, but not A11, accumulate to a 2-4-fold higher level than in hermaphrodite populations, suggesting that these transcripts are expressed at a higher level in males. The short transcripts are not male-specific, however, since they are also expressed in hermaphrodites. Thus, the A11 and D01 cDNA clones represent detectable mRNAs that are expressed during larval and adult stages when DBL-1 is active in regulating body size and male tail patterning.

**Table 1:** Relative expression levels of *sma-9 *transcript variants

Strain	Developmental Timepoint	A11 Transcript Level	D01 Transcript Level
N2	L1	10.12 ± 1.06	86.35 ± 1.17

N2	L3	8.88 ± 2.58	45.00 ± 0.87

N2	adult	8.97 ± 0.43	84.58 ± 7.82

*him-5*	L1	8.23 ± 0.28	87.01 ± 0.37

*him-5*	L3	2.18 ± 0.00	37.73 ± 0.11

*sma-9(qc3);him-5*	L3	6.20 ± 0.01	49.55 ± 1.29

*sma-9(wk55);him-5*	L3	1.81 ± 0.09	88.50 ± 1.32

*sma-9(cc604);him-5*	L3	9.40 ± 0.05	62.70 ± 1.26

Mean expression levels are give as percent of the expression level of exon-9-containing control, plus or minus standard deviation.

### Loss of function of sma-9 variant transcripts

Previous work has shown that *sma-9 *constructs lacking the Zn finger triplet (ZF3-5) are unable to rescue the body size and mesodermal patterning defects of a *sma-9 *mutant [14]. These experiments did not test, however, for differential requirements of short vs. full-length transcripts. To test whether *sma-9 *variant transcripts have specific functional roles, we performed RNAi targeting three different regions of the *sma-9 *gene. In previous experiments, dsRNA was introduced by microinjection [13,14], but we have found that RNAi by feeding results in a higher penetrance of male tail defects. We have therefore repeated these experiments using the feeding technique [29], and extended them by targeting the internal exon 9 in addition to the 5' and 3' ends of the gene. The effectiveness of each RNAi treatment was monitored by the appearance of the characteristic small body size phenotype (data not shown).

As a component in the DBL-1 pathway, *sma-9 *mutant phenotypes include small body size and male abnormal phenotypes [13]. One aspect of the male abnormal phenotype in *sma-9 *is the fusion of male tail sensory rays 8 and 9, two of the nine bilaterally symmetrical pairs of male-specific sensory organs. In wild-type animals, ray 8-9 fusions are also observed, but at a lower frequency. Treatment of wild-type animals with RNAi targeting exons 1-7 caused no increase in the frequency of ray 8-9 fusions (Table 2), although the small body size phenotype was produced. This treatment is predicted to knockdown expression of full-length and A11 variants but not the D01 variant. Conversely, RNAi targeting exons 21-25, predicted to knockdown the D01 and full-length variants, resulted in a two-fold increase in the frequency of ray 8-9 fusions (Table 2). These results are consistent with our previous findings that RNAi of the 3' end of the gene results in more severe male abnormal phenotypes than inactivation of the 5' end [13]. We next compared the male tail phenotype produced by RNAi targeting the central exon 9, predicted to inhibit full-length transcripts but not the short transcripts. This treatment resulted in a male tail defect of lower penetrance than that produced by RNAi targeting the 3' end (Table 2). Since the most severe male tail defect occurred when D01 was targeted, these experiments suggest that D01 has a role in regulating sensory ray patterning. Full-length *sma-9 *products are also predicted to function in sensory ray patterning, since the inhibition of a central exon resulted in a partial male abnormal phenotype.

**Table 2:** *sma-9 *loss of function phenotypes in male tail patterning and body size

RNAi (Exon) or Mutant	Forms Predicted to be Blocked	**Frequency of Ray 8-9 Fusions**^**1 **^**(n)**	**Body Length**^**2 **^**(n)**
none	none	18% (50)	ND

1-7	full-length, A11	20% (50)	ND

9	full-length only	29% (100)	ND

21-25	full-length, D01	36% (50)	ND

*sma-9(qc3)*	full-length, A11	50% (50)	692 ± 85 (43)

*sma-9(wk55)*	full-length only	66% (50)	566 ± 34 (31)

*sma-9(cc604)*	full-length, D01	72% (50)	716 ± 61 (30)

^1^All male tail phenotypes are scored with *him-5(e1490) *in the background to increase the frequency of males.

^2^Body length is given in μm ± standard deviation at 96 hours after embryo collection.

ND: not determined.

**Figure 3:**
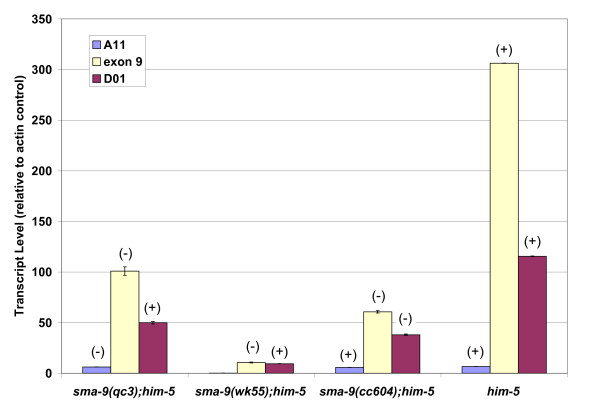
**Expression levels of *sma-9 *transcripts in mutant backgrounds**. Expression levels of *sma-9 *transcripts as determined by qRT-PCR. Animals were collected at the L3 stage. Data are shown as transcript abundance relative to *act-1 *actin gene control, and error bars show standard deviation. See Table 1 for quantitation of transcript abundance relative to exon-9-containing transcripts. (-): transcript harbors a premature termination codon. (+): transcript sequence is normal.

We sought to repeat this analysis using genetic mutants, since the RNAi treatments might cause some nonspecific effects due to targeting of unspliced pre-mRNA or to RNAi spreading [30,31]. We took advantage of three *sma-9 *alleles containing premature termination codons at different locations in the gene (Figure 1; [13,14]): *qc3 *in predicted exon 1 (within A11), *wk55 *in predicted exon 9 (between the short variants), and *cc604 *in predicted exon 18 (within D01). Therefore, in *qc3*, only the D01 transcript is capable of encoding a functional gene product; in *wk55*, both A11 and D01, but not the full-length transcript, are capable of encoding functional gene products; and in *cc604*, only the A11 transcript is capable of encoding a functional gene product. Since additional disruptions may occur due to nonsense-mediated decay [32], we determined the transcript expression profile for these strains at the L3 stage (Figure 3; Table 1). In all three mutant backgrounds, the transcript levels are somewhat reduced compared to the *him-5 *control. The reduction in transcript levels is most striking in the *sma-9(wk55) *background (Figure 3). This severe reduction in transcript levels is consistent with our observations that this allele causes the most severe body size phenotype (Table 2; [13]). Most importantly for the interpretation of our experiments, *sma-9(qc3) *and *sma-9(cc604) *animals express the short transcripts at detectable levels near the normal range (Figure 3).

The usual expectation for a series of nonsense mutations is that the earliest premature termination codon will have the most severe mutant phenotype while later termination codons may cause less severe defects if those alleles encode partially functional gene products. For the *sma-9 *alleles, the opposite result is seen in the male tail. The allele encoding the latest termination codon, *cc604*, causes the most severe male tail defect, with a frequency of ray 8-9 fusions greater than any of the previously characterized alleles (Table 2, [13]). Since different investigators typically vary in their quantitation of ray fusion frequencies, we also repeated measurement of the male tail defects of *qc3 *and *wk55 *for direct comparison. Consistent with previous results, *qc3*, the allele containing the earliest termination codon, causes a mild male tail defect, while *wk55*, containing a termination codon in exon 9, results in an intermediate frequency of ray fusions (Table 2). Thus, the severity of the sensory ray defect is inversely correlated with predicted D01 activity. The most severe defect is manifested in *cc604 *mutants, in which the D01 isoform is disrupted by mutation. An intermediate frequency of ray fusions occurs in *wk55 *mutants, in which the D01 transcript is expressed at reduced levels but is not disrupted by mutation. A mild male tail defect is seen in *qc3 *mutants, in which levels of D01 transcripts are lower than in wild type but higher than in *wk55 *(Figure 3). Overall, with regard to male tail patterning, these mutations form an allelic series in which their levels of activity are the opposite of that expected.

In body size, a different result was obtained with these three mutants (Table 2). The *sma-9(wk55) *mutant shows the smallest body size. In contrast, both *qc3 *and *cc604 *produce less severe body length phenotypes that are mutually indistinguishable (Table 2). Since *qc3 *and *cc604 *produce indistinguishable body size defects, this analysis does not support a specific role for either the A11 or the D01 transcript in the regulation of body size. Furthermore, the more severe body size phenotype of *wk55 *mutants could be due to the lower overall level of *sma-9 *expression in these mutants (Figure 3). The results of body size analysis thus suggest that full-length isoforms, rather than A11 or D01, are critical for promoting growth. Based on our RNAi data and on the differential effects of the three nonsense alleles on body size and male tail development, we hypothesize that the D01 short transcript has a tissue-specific function in sensory ray development.

### Rescue of sma-9 mutant phenotypes by overexpression of D01 cDNA

**Figure 4:**
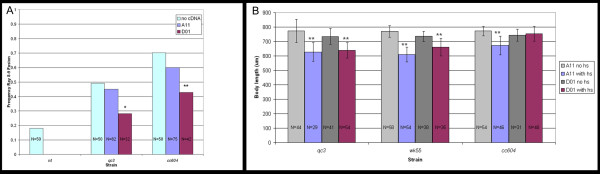
**Phenotypic rescue by overexpression of *sma-9 *short transcripts**. **A**. Male tail phenotypes are given as frequency of fusion of rays 8 and 9 per male tail side scored. p values were calculated using a Student's t-test. *: significant difference from no cDNA control (p < 0.05). **: highly significant difference from no cDNA control (p < 0.01). The p values for experiments with A11 cDNA overexpression showed no significant difference from controls (p = 0.59 for *qc3*; p = 0.16 for *cc604*). All strains contain *him-5(e1490) *to increase the proportion of males. **B**. Body size phenotypes. Mean body length at 96 hours after embryo collection (adult stage) is shown. Error bars show standard deviation. Note that these strains are transgenics expressing the *rol-6 *marker that influences body length, so all comparisons are made between heat-shocked animals and no heat shock control rather than using a nontransgenic control. p values were calculated using a Student's t-test. **: highly significant difference from no heat shock control (p < 0.01).

Consistent with our hypothesis, we have previously demonstrated that overexpression of the D01 cDNA, but not of A11, from a heat shock promoter can partially rescue *sma-9(wk55) *mutant male tail defects [33]. In comparison, expression of *sma-9 *from a cosmid genomic clone containing the entire coding region resulted in nearly complete rescue of the ray fusion defect [13]. We next asked whether expression of *sma-9 *short transcripts is sufficient to provide normal gene function in *qc3 *and in *cc604 *mutants, in which the A11 and the D01 transcript, respectively, are disrupted by premature termination codons. As before, transgenics carrying *hs-sma-9 *constructs were subjected to heat shock during the L3 stage when sensory ray identities are being established [34] and males were scored in adulthood. In *cc604 *mutants, which contain a premature termination codon within the D01 transcript, expression of D01 resulted in partial rescue of the male tail defect (Figure 4A). In contrast, overexpression of A11 in *cc604 *caused a slight reduction in frequency of sensory ray fusions, but this effect was not statistically significant (p = 0.16). In *qc3 *mutants, in which the A11 transcript contains a premature termination codon, we were surprised to find that overexpression of A11 produced no change in male tail phenotype, whereas overexpression of D01 resulted in significant rescue of the male tail defect (Figure 4A). Based on the rescue of male tail defects by D01 in both *qc3 *and *cc604 *mutants, we conclude that increased expression of D01 may be able to compensate partially for defective full-length *sma-9 *transcripts.

Finally, we looked for evidence of A11 or D01 function in body size regulation. The body size phenotypes of *qc3 *and *cc604 *did not support a specific role for either A11 or D01 in body size regulation, since these two mutants have indistinguishable sizes (Table 2). To determine whether increased expression of A11 and/or D01 can promote growth, we used our heat shock constructs to drive their expression. Heat shock of transgenics in the L3 stage produced no evident changes in body size (data not shown), so we performed heat shock earlier in development, in L1 animals, and measured body length in adulthood. In contrast to the rescue of male abnormal phenotypes by D01, heat shock of A11 and D01 transgenics led either to a reduction in body size or to no change in body size (Figure 4B). These results suggest that A11 and D01 do not contribute to the growth promoting activity of *sma-9*. If anything, increased expression of A11 and D01 may interfere with the ability of gene products from full-length transcripts to promote increases in body size.

## Discussion

We have shown here that the *C. elegans sma-9/schnurri *locus undergoes a novel *trans*-splicing process to generate two shorter transcripts, one of which has a tissue-specific function in DBL-1/BMP signaling. First, we have demonstrated that an SL2 *trans*-spliced, truncated cDNA form, *yk1237d01 *(D01), represents an actual mRNA that is detectable by RT-PCR. Second, we have verified that longer transcripts containing both the 5' and 3' ends of the gene are also detectable by RT-PCR. Third, loss-of-function experiments (RNAi and premature termination mutants) demonstrate that the short transcript D01 is necessary but not sufficient for male tail patterning, since depletion of both D01 and full-length transcripts causes a more severe male tail patterning defect than depletion of full-length transcripts alone. Fourth, gain-of-function experiments (heat shock-induced overexpression) indicate that the short transcript D01 can partially rescue male tail but not body size defects of *sma-9 *mutants. In contrast to the results on D01, we find for the *yk1285a11 *(A11) transcript little or no evidence of an *in vivo *function, as well as low abundance throughout development. In addition, class I and II full-length transcripts and the D01 short transcript, but not the A11 transcript, encode the Zn finger triplet domain shown to be critical in the regulation of body size and mesodermal patterning [14]. In *Drosophila*, this domain is also critical in mediating Dpp-responsive target gene regulation [35]. The gene product encoded by the A11 transcript lacks this domain and may therefore not be capable of being recruited to target genes. Why then is the A11 transcript detected at all? One possibility is that it is generated as a byproduct of the *trans*-splicing mechanism that generates D01.

Since we have shown that full-length transcripts and D01 transcripts are each necessary but not sufficient for male tail patterning, we must consider what differences in molecular function are found in the respective gene products. In other organisms, Schnurri homologs have been shown to act either as transcriptional activators [20,21,36] or as transcriptional repressors [16,37] mediating BMP-dependent transcription. Strikingly, Yao et al. [21] have shown by swapping *Drosophila *and Xenopus Schnurri homologs that transcriptional activation or repression activities may be context-dependent rather than an intrinsic property of the protein. In *C*. *elegans*, we have previously analyzed intrinsic transcriptional activities of SMA-9 protein domains using a heterologous transcription assay [33]. In these experiments, the acidic residue-rich domain (ARD) in exon 14 exhibited transcriptional activator activity, while the N-terminal region of SMA-9 displayed intrinsic transcriptional repressor activity [33]. Full-length *sma-9 *transcripts should encode protein products containing both of these transcriptional domains, so that their transcriptional activities may be context-dependent as was described for *Drosophila *and Xenopus Schnurri [21]. On the other hand, D01 transcripts encode products containing solely the transcriptional activation domain (Figure 1). We therefore hypothesize that full-length SMA-9 isoforms function as transcriptional repressors or activators depending on context, while D01-encoded isoforms may be obligate transcriptional activators. This transcriptional activation activity may be necessary for the robust activation of *sma-9 *target genes in sensory ray development, but may be dispensable in body size regulation. Consistent with this hypothesis, a SMA-9 Zn finger domain fusion with a known transcriptional activator, VP16, can partially rescue the male tail defects but not the body size defects of *sma-9 *mutants. In contrast to the SMA-9::VP16 fusion, a SMA-9::enR repression domain fusion rescues body size, suggesting that transcriptional repression is more important than activation for SMA-9 activity in body size regulation [33]. Overall, the *in vivo *activity of the SMA-9::VP16 fusion is strikingly similar to that of the D01 isoform.

Alternative splicing is regulated by *trans*-acting factors that influence the choice of splice sites. It will be of interest to determine the *trans-*acting factors that regulate alternative *trans-*splicing of *sma-9*. In preliminary experiments, we tested whether *sma-9 trans-*splicing depends on *mec-8*, which encodes an RNA recognition motif-containing protein that regulates alternative splicing in the hypodermis [38,39]. We found that A11, D01, and exon 9-containing transcript levels were all reduced 2- to 5-fold in *mec-8 *mutants relative to N2 (data not shown). If *mec-8 *were directly involved in processing the short transcripts from a longer precursor, we expected to find reciprocal changes in accumulation of the full-length transcripts relative to short transcripts. Our results are therefore not sufficiently clear to draw a conclusion as to whether MEC-8 is directly involved in *sma-9 trans-*splicing, and additional investigations will be necessary to identify the *trans-*acting factors that mediate this reaction.

A variety of genetic mechanisms exist for generating diverse functions from a single genetic locus, including those that affect transcript structure such as alternative splicing, alternative transcriptional start sites, and alternative polyadenylation. One prominent example of alternative splicing is the regulation of sex determination in *Drosophila *by Sex-lethal [40]. This system also uses developmentally regulated alternative promoters. In *C. elegans*, extensive use of alternative promoters leading to different protein isoforms has been documented [41]. Alternative polyadenylation can also lead to the production of truncated protein variants, such as in the *daf-4 *BMP receptor gene in *C. elegans*, in which alternative polyadenylation leads to the production of a secreted negative inhibitor of DAF-4 signaling [42]. To our knowledge, alternative *trans*-splicing as we have characterized it for *sma-9 *has not previously been reported for other loci in *C. elegans*. The unique feature of this *trans*-splicing event is the choice between processing the transcript via *cis- *or *trans-*splicing at the splice acceptor site of predicted exon 11. In an organism without *trans*-splicing, a similar truncation could be achieved via use of a downstream transcriptional start site or via proteolytic cleavage. For example, in the Hedgehog signaling pathway, full-length transcription factors of the Ci/Gli family are transcriptional activators that, upon proteolytic cleavage, are converted to transcriptional repressors [43]. We speculate that since the *trans*-splicing machinery exists in *C. elegans*, it was available to be recruited for this unusual role in the *sma-9 *locus.

## Conclusions

Both *C. elegans sma-9/schnurri *[13] and the human homologs Shn-1 and Shn-3 [23,24] display alternative splicing that is predicted to result in multiple protein isoforms. For most of these isoforms, however, no specific *in vivo *function has been described. We have now demonstrated a functional role for an alternatively *trans-*spliced *sma-9 *short transcript represented by cDNA clone *yk1237d01 *(D01). This transcript mediates a tissue-specific role, since it is required for sensory ray development but not for body size regulation. Our results provide insight into how a single genetic locus can contribute to diverse protein functions.

## Methods

### C. elegans strains and culture

Nematodes were cultured using standard methods and grown at 20°C unless otherwise noted [44]. In addition to strains generated in this work, the following strains were used: N2 (wild type); LG V, *him-5(e1490)*; LG X, *sma-9(qc3, wk55, cc604)*.

### RT-PCR

RT-PCR was performed in two steps using SuperScript III First-Strand Synthesis System (Invitrogen) and RNA extracted from mixed-stage N2 wild-type animals. Temperature gradient PCR was performed on an Eppendorf gradient thermal cycler (MasterCycler) following the manufacturer's instructions.

Real time RT-PCR was performed on a LightCycler 2.0 (Roche). Data analysis software used was LightCycler software version 4.0. Animals were collected at desired time points and total RNA extracted by Trizol as described [13]. SuperScriptTM III platinum two-step qRT-PCR kit with SYBR Green (Invitrogen) was used to perform RT-PCR. Actin gene *act-1 *was used as standard control. A primer in an intron of the *act-1 *gene was used to confirm the absence of genomic DNA in RNA preps. Primer sequences for all RT-PCR experiments are available upon request.

### RNAi

RNAi feeding method was performed as described [29] after cloning *sma-9 *fragments into RNAi feeding vector pPD129.36 (a gift from Dr. A. Fire). To score sensory ray patterning, only adult males displaying a small phenotype were collected and scored to ensure that the animals analyzed had undergone a knockdown of *sma-9 *activity.

### Heat-shock clone constructs and generation of transgenic animals

Two *sma-9 *splice variants *yk1285a11 *and *yk1237d01 *(GenBank Accession Numbers: AY390537 and AY390550) were identified previously [13], which were SL1- and SL2- *trans*-spliced cDNA clones that appeared to represent shorter but complete mRNAs. Heat-shock vector pPD49.83 was a kind gift from Dr. J. Liu. *yk1285a11 *and *yk1237d01 *were cloned into pPD49.83 at Nhe I and Kpn I sites. Transgenic nematodes were generated by microinjection of constructs (10 ng/ul) into the gonadal syncytia of him-5 hermaphrodites, with *rol-6 *(100 ng/ul) as a marker [45]. Mutant strains carrying transgenic constructs were generated by appropriate crosses between the mutant strains and transgenic lines.

### Heat-shock experiments

Gravid hermaphrodites were washed off plates with M9 buffer and collected in an Eppendorf tube. Hermaphrodites were destroyed with sodium hypochlorite solution, leaving eggs that were collected and inoculated onto OP50-seeded plates. For body size measurement, 24 hours after egg collection, worms were collected and transferred to a siliconized Eppendorf tube containing 100 ul of M9 buffer [44], which was placed in a circulating water bath under the heat-shock conditions specified. After heat-shock, worms were recovered, placed at 25°C on OP50-seeded plates and allowed to develop to adulthood. The length of individual worms was measured using Image-Pro Express software (Sigma) with Nomarski optics. For male tail ray examination, worms were collected 72 hours after egg collection and transferred to a siliconized Eppendorf tube containing 100 ul of M9 buffer [44], which was placed in a circulating water bath under the heat-shock conditions specified. After heat-shock, worms were recovered, placed at 25°C on OP50-seeded plates and allowed to develop to adulthood and then scored for ray patterning.

## Authors' contributions

JY performed the heat shock and RT-PCR experiments and participated in interpretation of data. LY performed the RNAi experiments. CSD conceived of the study, participated in its design and coordination, analyzed mutant phenotypes, and drafted the manuscript. All authors read and approved the final manuscript.

## References

[B1] Roberts AB Sporn MB Physiological actions and clinical applications of transforming growth factor-beta (TGF-beta) Growth Factors 1993 8 1 1 9 10.3109/08977199309029129 8448037

[B2] Massague J Chen YG Controlling TGF-beta signaling Genes Dev 2000 14 6 627 644 10733523

[B3] Wrana JL Attisano L Carcamo J Zentella A Doody J Laiho M Wang XF Massague J TGF beta signals through a heteromeric protein kinase receptor complex Cell 1992 71 6 1003 1014 10.1016/0092-8674(92)90395-S 1333888

[B4] Liu F Ventura F Doody J Massague J Human type II receptor for bone morphogenic proteins (BMPs): extension of the two-kinase receptor model to the BMPs Mol Cell Biol 1995 15 7 3479 3486 7791754 10.1128/mcb.15.7.3479 PMC230584

[B5] Inman GJ Nicolas FJ Hill CS Nucleocytoplasmic shuttling of Smads 2, 3, and 4 permits sensing of TGF-beta receptor activity Mol Cell 2002 10 2 283 294 10.1016/S1097-2765(02)00585-3 12191474

[B6] Macias-Silva M Abdollah S Hoodless PA Pirone R Attisano L Wrana JL MADR2 is a substrate of the TGFbeta receptor and its phosphorylation is required for nuclear accumulation and signaling Cell 1996 87 7 1215 1224 10.1016/S0092-8674(00)81817-6 8980228

[B7] Kretzschmar M Liu F Hata A Doody J Massague J The TGF-beta family mediator Smad1 is phosphorylated directly and activated functionally by the BMP receptor kinase Genes Dev 1997 11 8 984 995 10.1101/gad.11.8.984 9136927

[B8] Wu RY Zhang Y Feng XH Derynck R Heteromeric and homomeric interactions correlate with signaling activity and functional cooperativity of Smad3 and Smad4/DPC4 Mol Cell Biol 1997 17 5 2521 2528 9111321 10.1128/mcb.17.5.2521 PMC232101

[B9] Zhang Y Feng X We R Derynck R Receptor-associated Mad homologues synergize as effectors of the TGF-beta response Nature 1996 383 6596 168 172 10.1038/383168a0 8774881

[B10] Inman GJ Hill CS Stoichiometry of active smad-transcription factor complexes on DNA J Biol Chem 2002 277 52 51008 51016 10.1074/jbc.M208532200 12374795

[B11] Lagna G Hata A Hemmati-Brivanlou A Massague J Partnership between DPC4 and SMAD proteins in TGF-beta signalling pathways Nature 1996 383 6603 832 836 10.1038/383832a0 8893010

[B12] Savage-Dunn C The C. elegans Research Community TGF-β signaling WormBook 2005 9 1 12 10.1895/wormbook.1.22.1 18050404 PMC4781025

[B13] Liang J Lints R Foehr ML Tokarz R Yu L Emmons SW Liu J Savage-Dunn C The Caenorhabditis elegans schnurri homolog sma-9 mediates stage- and cell type-specific responses to DBL-1 BMP-related signaling Development 2003 130 26 6453 6464 10.1242/dev.00863 14627718

[B14] Foehr ML Lindy AS Fairbank RC Amin NM Xu M Yanowitz J Fire AZ Liu J An antagonistic role for the C. elegans Schnurri homolog SMA-9 in modulating TGFbeta signaling during mesodermal patterning Development 2006 133 15 2887 2896 10.1242/dev.02476 16790477

[B15] Dai H Hogan C Gopalakrishnan B Torres-Vazquez J Nguyen M Park S Raftery LA Warrior R Arora K The zinc finger protein schnurri acts as a Smad partner in mediating the transcriptional response to decapentaplegic Dev Biol 2000 227 2 373 387 10.1006/dbio.2000.9901 11071761

[B16] Marty T Muller B Basler K Affolter M Schnurri mediates Dpp-dependent repression of brinker transcription Nat Cell Biol 2000 2 10 745 749 10.1038/35036383 11025666

[B17] Takagi T Harada J Ishii S Murine Schnurri-2 is required for positive selection of thymocytes Nat Immunol 2001 2 11 1048 1053 10.1038/ni728 11668343

[B18] Oukka M Kim ST Lugo G Sun J Wu LC Glimcher LH A mammalian homolog of Drosophila schnurri, KRC, regulates TNF receptor-driven responses and interacts with TRAF2 Mol Cell 2002 9 1 121 131 10.1016/S1097-2765(01)00434-8 11804591

[B19] Wu LC ZAS: C2H2 zinc finger proteins involved in growth and development Gene Expr 2002 10 4 137 152 12173742 10.3727/000000002783992479 PMC5977514

[B20] Jin W Takagi T Kanesashi SN Kurahashi T Nomura T Harada J Ishii S Schnurri-2 controls BMP-dependent adipogenesis via interaction with Smad proteins Dev Cell 2006 10 4 461 471 10.1016/j.devcel.2006.02.016 16580992

[B21] Yao LC Blitz IL Peiffer DA Phin S Wang Y Ogata S Cho KW Arora K Warrior R Schnurri transcription factors from Drosophila and vertebrates can mediate Bmp signaling through a phylogenetically conserved mechanism Development 2006 133 20 4025 4034 10.1242/dev.02561 17008448

[B22] Shukla A Malik M Cataisson C Ho Y Friesen T Suh KS Yuspa SH TGF-beta signalling is regulated by Schnurri-2-dependent nuclear translocation of CLIC4 and consequent stabilization of phospho-Smad2 and 3 Nat Cell Biol 2009 11 6 777 784 10.1038/ncb1885 19448624 PMC2825893

[B23] Muchardt C Seeler JS Nirula A Shurland DL Gaynor RB Regulation of human immunodeficiency virus enhancer function by PRDII-BF1 and c-rel gene products J Virol 1992 66 1 244 250 1727488 10.1128/jvi.66.1.244-250.1992 PMC238281

[B24] Hicar MD Liu Y Allen CE Wu LC Structure of the human zinc finger protein HIVEP3: molecular cloning, expression, exon-intron structure, and comparison with paralogous genes HIVEP1 and HIVEP2 Genomics 2001 71 1 89 100 10.1006/geno.2000.6425 11161801

[B25] Blumenthal T The C. elegans Research Community Trans-splicing and operons WormBook 2005 25 1 9 10.1895/wormbook.1.5.1 18050426

[B26] Huang T Kuersten S Deshpande AM Spieth J MacMorris M Blumenthal T Intercistronic region required for polycistronic pre-mRNA processing in Caenorhabditis elegans Mol Cell Biol 2001 21 4 1111 1120 10.1128/MCB.21.4.1111-1120.2001 11158298 PMC99565

[B27] Blumenthal T Evans D Link CD Guffanti A Lawson D Thierry-Mieg J Thierry-Mieg D Chiu WL Duke K Kiraly M A global analysis of Caenorhabditis elegans operons Nature 2002 417 6891 851 854 10.1038/nature00831 12075352

[B28] Lamesch P Milstein S Hao T Rosenberg J Li N Sequerra R Bosak S Doucette-Stamm L Vandenhaute J Hill DE C. elegans ORFeome version 3.1: increasing the coverage of ORFeome resources with improved gene predictions Genome Res 2004 14 10B 2064 2069 10.1101/gr.2496804 15489327 PMC528921

[B29] Kamath RS Ahringer J Genome-wide RNAi screening in Caenorhabditis elegans Methods 2003 30 4 313 321 10.1016/S1046-2023(03)00050-1 12828945

[B30] Sijen T Fleenor J Simmer F Thijssen KL Parrish S Timmons L Plasterk RH Fire A On the role of RNA amplification in dsRNA-triggered gene silencing Cell 2001 107 4 465 476 10.1016/S0092-8674(01)00576-1 11719187

[B31] Bosher JM Dufourcq P Sookhareea S Labouesse M RNA interference can target pre-mRNA: consequences for gene expression in a Caenorhabditis elegans operon Genetics 1999 153 3 1245 1256 10545456 10.1093/genetics/153.3.1245 PMC1460805

[B32] Mango SE Stop making nonSense: the C. elegans smg genes Trends Genet 2001 17 11 646 653 10.1016/S0168-9525(01)02479-9 11672865

[B33] Liang J Yu L Yin J Savage-Dunn C Transcriptional repressor and activator activities of SMA-9 contribute differentially to BMP-related signaling outputs Dev Biol 2007 305 2 714 725 10.1016/j.ydbio.2007.02.038 17397820

[B34] Lints R Emmons SW Patterning of dopaminergic neurotransmitter identity among Caenorhabditis elegans ray sensory neurons by a TGFbeta family signaling pathway and a Hox gene Development 1999 126 24 5819 5831 10572056 10.1242/dev.126.24.5819

[B35] Muller B Hartmann B Pyrowolakis G Affolter M Basler K Conversion of an extracellular Dpp/BMP morphogen gradient into an inverse transcriptional gradient Cell 2003 113 2 221 233 10.1016/S0092-8674(03)00241-1 12705870

[B36] Torres-Vazquez J Park S Warrior R Arora K The transcription factor Schnurri plays a dual role in mediating Dpp signaling during embryogenesis Development 2001 128 9 1657 1670 11290303 10.1242/dev.128.9.1657

[B37] Pyrowolakis G Hartmann B Muller B Basler K Affolter M A simple molecular complex mediates widespread BMP-induced repression during Drosophila development Dev Cell 2004 7 2 229 240 10.1016/j.devcel.2004.07.008 15296719

[B38] Spike CA Davies AG Shaw JE Herman RK MEC-8 regulates alternative splicing of unc-52 transcripts in C. elegans hypodermal cells Development 2002 129 21 4999 5008 12397108 10.1242/dev.129.21.4999

[B39] Lundquist EA Herman RK Rogalski TM Mullen GP Moerman DG Shaw JE The mec-8 gene of C. elegans encodes a protein with two RNA recognition motifs and regulates alternative splicing of unc-52 transcripts Development 1996 122 5 1601 1610 8625846 10.1242/dev.122.5.1601

[B40] Black DL Mechanisms of alternative pre-messenger RNA splicing Annu Rev Biochem 2003 72 291 336 10.1146/annurev.biochem.72.121801.161720 12626338

[B41] Choi J Newman AP A two-promoter system of gene expression in C. elegans Dev Biol 2006 296 2 537 544 10.1016/j.ydbio.2006.04.470 16765937

[B42] Gunther CV Riddle DL Alternative polyadenylation results in a truncated daf-4 BMP receptor that antagonizes DAF-7-mediated development in Caenorhabditis elegans J Biol Chem 2004 279 38 39555 39564 10.1074/jbc.M407602200 15254038

[B43] Huangfu D Anderson KV Signaling from Smo to Ci/Gli: conservation and divergence of Hedgehog pathways from Drosophila to vertebrates Development 2006 133 1 3 14 10.1242/dev.02169 16339192

[B44] Brenner S The genetics of Caenorhabditis elegans Genetics 1974 77 1 71 94 4366476 10.1093/genetics/77.1.71 PMC1213120

[B45] Mello CC Kramer JM Stinchcomb D Ambros V Efficient gene transfer in C.elegans: extrachromosomal maintenance and integration of transforming sequences Embo J 1991 10 12 3959 3970 1935914 10.1002/j.1460-2075.1991.tb04966.x PMC453137

